# Supplementary material for: Consonant and vowel articulation accuracy in younger and middle-aged Spanish healthy adults
Source: PLoS One. 2020 Nov 9;15(11):e0242018. doi: 10.1371/journal.pone.0242018 (PMC7652263; doi:10.1371/journal.pone.0242018)
Supplement: S2 File — (DOCX) [file pone.0242018.s005.docx]

| N | Utterance | Type | List |
| --- | --- | --- | --- |
| 1 | ʝa te | W | 1 |
| 2 | no lu | NW | 1 |
| 3 | ba fe | NW | 1 |
| 4 | ti tu lo | W | 1 |
| 5 | mo de | NW | 1 |
| 6 | pe no | NW | 1 |
| 7 | xe ɾa | NW | 1 |
| 8 | pa le ti ʝa | W | 1 |
| 9 | ka ro fa | NW | 1 |
| 10 | ma po | NW | 1 |
| 11 | ʧu lo | W | 1 |
| 12 | pa ba ɾi mo | NW | 1 |
| 13 | ko pa di | NW | 1 |
| 14 | li ga | W | 1 |
| 15 | be ʝo lu | NW | 1 |
| 16 | so te to | NW | 1 |
| 17 | ma ɾe xa θa | NW | 1 |
| 18 | nu me ɾo | W | 1 |
| 19 | ka pi tu bo | NW | 1 |
| 20 | bi ka | NW | 1 |
| 21 | ti bo | NW | 1 |
| 22 | ka ʝi | NW | 1 |
| 23 | fo bo xe ma | NW | 1 |
| 24 | pa ne | NW | 1 |
| 25 | tu fo | W | 1 |
| 26 | sa te li to | NW | 1 |
| 27 | fe ʧa | W | 1 |
| 28 | pe mi | NW | 1 |
| 29 | di ne so | W | 1 |
| 30 | ka ba ʝe te | W | 1 |
| 31 | ko li θa | NW | 1 |
| 32 | ka ro ma do | NW | 1 |
| 33 | gi ɲo | W | 1 |
| 34 | ma ɲa da | NW | 1 |
| 35 | θa fe lu di | NW | 1 |
| 36 | la ɾo | NW | 1 |
| 37 | θi ne | W | 1 |
| 38 | se ɲo ɾi ta | W | 1 |
| 39 | no fe | NW | 1 |
| 40 | pe li ku la | W | 1 |
| 41 | ku xo ba | NW | 1 |
| 42 | fu xo | NW | 1 |
| 43 | se ku | NW | 1 |
| 44 | to re | W | 1 |
| 45 | do fe | NW | 1 |
| 46 | ba re ɲo | W | 1 |
| 47 | me la ni to | NW | 1 |
| 48 | ta le go | W | 1 |
| 1 | so ʝa | NW | 2 |
| 2 | be ta | NW | 2 |
| 3 | sa te li te | W | 2 |
| 4 | ri bu po | NW | 2 |
| 5 | me po | NW | 2 |
| 6 | ma ɲa na | W | 2 |
| 7 | θi da | NW | 2 |
| 8 | ka ro ɲa | W | 2 |
| 9 | bi ga | W | 2 |
| 10 | mo pe di ta | NW | 2 |
| 11 | me θe do ta | NW | 2 |
| 12 | ko li ʝa | W | 2 |
| 13 | bu de | NW | 2 |
| 14 | mo ku | NW | 2 |
| 15 | lo ɾa | NW | 2 |
| 16 | mi ɾi θa | NW | 2 |
| 17 | xe ta | W | 2 |
| 18 | bu fe lu | NW | 2 |
| 19 | ma ni be θa | NW | 2 |
| 20 | ma ɾe xa da | W | 2 |
| 21 | pe so | W | 2 |
| 22 | di ɾa ni | NW | 2 |
| 23 | ti fe du ne | NW | 2 |
| 24 | ti ʝo | NW | 2 |
| 25 | no fe | NW | 2 |
| 26 | pe ta ti | NW | 2 |
| 27 | la do | W | 2 |
| 28 | mu no | NW | 2 |
| 29 | θa lu | NW | 2 |
| 30 | ka ro ma to | W | 2 |
| 31 | mu si ma | NW | 2 |
| 32 | de ba ti | NW | 2 |
| 33 | di ne ɾo | W | 2 |
| 34 | ni fu ba mi | NW | 2 |
| 35 | mo te | W | 2 |
| 36 | ko da | NW | 2 |
| 37 | ko θi | NW | 2 |
| 38 | di ku li te | NW | 2 |
| 39 | ko pa te | NW | 2 |
| 40 | do bu | NW | 2 |
| 41 | no ʧe | W | 2 |
| 42 | fi lo so θo | NW | 2 |
| 43 | pa do | NW | 2 |
| 44 | ka ʝo | W | 2 |
| 45 | ka pi tu lo | W | 2 |
| 46 | lu xa | NW | 2 |
| 47 | ba ʝe | W | 2 |
| 48 | ka ba ʝe mo | NW | 2 |
| 1 | so ɾi | NW | 3 |
| 2 | ʝe da | NW | 3 |
| 3 | pa li xo | NW | 3 |
| 4 | so ga | W | 3 |
| 5 | ko pa | W | 3 |
| 6 | be ʝa fe | NW | 3 |
| 7 | mu lo | W | 3 |
| 8 | θi ma | W | 3 |
| 9 | no be ʝa | NW | 3 |
| 10 | ni gu | NW | 3 |
| 11 | ku fe | NW | 3 |
| 12 | so ma | NW | 3 |
| 13 | ka ba ʝe ɾo | W | 3 |
| 14 | be na | W | 3 |
| 15 | mu si ka | W | 3 |
| 16 | di ku | NW | 3 |
| 17 | ka mi ɾo | NW | 3 |
| 18 | me θe do ɾa | W | 3 |
| 19 | θe bi | NW | 3 |
| 20 | ka xo | NW | 3 |
| 21 | bi la | NW | 3 |
| 22 | me di θi ɾa | NW | 3 |
| 23 | la ti ga fo | NW | 3 |
| 24 | no fe ba | NW | 3 |
| 25 | lu fo | NW | 3 |
| 26 | ka po di no | NW | 3 |
| 27 | fi lo so fo | W | 3 |
| 28 | ni bo fe ma | NW | 3 |
| 29 | mi ɾi ʝa | W | 3 |
| 30 | θi bu fe ni | NW | 3 |
| 31 | mo pa so ka | NW | 3 |
| 32 | ma ni be la | W | 3 |
| 33 | pe ta te | W | 3 |
| 34 | de ba te | W | 3 |
| 35 | lu ʧa | W | 3 |
| 36 | ka mo | NW | 3 |
| 37 | ko di so | NW | 3 |
| 38 | so ma θi | NW | 3 |
| 39 | lo na | W | 3 |
| 40 | ro ko | NW | 3 |
| 41 | pe ta ma | NW | 3 |
| 42 | ge θa | NW | 3 |
| 43 | ma no ta go | NW | 3 |
| 44 | bu ke | W | 3 |
| 45 | me pe | NW | 3 |
| 46 | pe ta | NW | 3 |
| 47 | re ti ɾa θa | NW | 3 |
| 48 | ti bu | NW | 3 |
| 1 | ʧu so | NW | 4 |
| 2 | fe θa | NW | 4 |
| 3 | ʝe ma | W | 4 |
| 4 | θi fo | NW | 4 |
| 5 | pa ni | NW | 4 |
| 6 | θa bo lu | NW | 4 |
| 7 | ma no ta θo | W | 4 |
| 8 | ba re ʝo | NW | 4 |
| 9 | se ɲo ɾi da | NW | 4 |
| 10 | tu θo | NW | 4 |
| 11 | pe ta ka | W | 4 |
| 12 | do ʝa | NW | 4 |
| 13 | li ʝa | NW | 4 |
| 14 | ka mi no | W | 4 |
| 15 | pa de so | NW | 4 |
| 16 | so da | W | 4 |
| 17 | ti tu mo | NW | 4 |
| 18 | di ma ko | NW | 4 |
| 19 | ka no | NW | 4 |
| 20 | ko ga | NW | 4 |
| 21 | mo pe | NW | 4 |
| 22 | na fe | NW | 4 |
| 23 | ge ra | W | 4 |
| 24 | so ga li na | NW | 4 |
| 25 | pe li ku θa | NW | 4 |
| 26 | di la | NW | 4 |
| 27 | no be la | W | 4 |
| 28 | pe ku ga ta | NW | 4 |
| 29 | la ti ga θo | W | 4 |
| 30 | ro ka | W | 4 |
| 31 | pa le ti ra | NW | 4 |
| 32 | θe bo | W | 4 |
| 33 | pa li ʝo | W | 4 |
| 34 | ka ba ʝe to | NW | 4 |
| 35 | lo fe du ne | NW | 4 |
| 36 | ʝa to | NW | 4 |
| 37 | gi ʧo | NW | 4 |
| 38 | pe na | W | 4 |
| 39 | bi ʝa | W | 4 |
| 40 | to ʝe | NW | 4 |
| 41 | fe bu xo | NW | 4 |
| 42 | re ti ɾa da | W | 4 |
| 43 | nu me so | NW | 4 |
| 44 | ta le bo | NW | 4 |
| 45 | me di θi na | W | 4 |
| 46 | θi se | NW | 4 |
| 47 | fe xi | NW | 4 |
| 48 | bi fu pe si | NW | 4 |
